# Veterinarians’ perceptions on African swine fever and the control measures in Estonia

**DOI:** 10.1186/s13028-025-00822-9

**Published:** 2025-08-27

**Authors:** Lidiia Moskalenko, Kerli Mõtus, Arvo Viltrop

**Affiliations:** Institute of Veterinary Medicine and Animal Sciences, Estonian University of Life Sciences, F.R.Kreutzwaldi, 51014 Tartu, Estonia

**Keywords:** Acceptability, ASF, Attitude, Awareness, Biosecurity, Domestic pig, Participatory epidemiology, Preventive measures, Control measures, Trust

## Abstract

**Background:**

Veterinarians are key stakeholders in fighting African Swine Fever (ASF), yet their awareness, perceptions and attitudes of ASF are often unknown. This is crucial, especially in Estonia where ASF has persisted for almost 10 years. We conducted five focus groups involving 11 farm veterinarians and 4 assistants working on Estonian commercial pig farms. Using participatory methods, we revealed their awareness of ASF signs, transmission routes, and preventive measures. Furthermore, we identified perceived obstacles to the implementation and maintenance of ASF biosecurity measures and their acceptance of control measures. Finally, we investigated veterinarians’ awareness of stakeholders in ASF control, their role and trust to fulfil these roles.

**Results:**

Haemorrhages on skin, mucosa and organs, along with fever, loss of appetite, and increased mortality were frequently mentioned as first signs that would lead veterinary staff to suspect ASF infection in the herd. The highest risk of virus introduction into the herd was designated to humans, transport vehicles, and bedding. Training of people and disinfection with movement restrictions were considered the most effective measures for preventing ASF. The motivation and attitude of farm employees, and financial constraints were perceived as major obstacles impacting implementation and maintenance of ASF biosecurity measures. Herd-level ASF eradication measures were generally accepted, except for culling. The majority acknowledged its necessity, while others advocated for a case-by-case approach or suggested using the animals for food. Establishing restricted zones I, II, and III received the least acceptance, with concerns over market access, product prices, economic hardships for farmers, and inconsistencies in zoning practices across the EU. Pigkeepers and veterinary authorities were seen as the key stakeholders in ASF control, with veterinarians and pigkeepers being the most trusted to fulfil their roles.

**Conclusions:**

Veterinary staff demonstrated good awareness of ASF signs, transmission routes, and preventive measures. Further training in ASF control measures remains relevant in areas related to the EU and national legislation, involved parties and their roles, to ensure effective implementation and collaboration with stakeholders. This study provides insights into refining ASF communication strategies and identifying potential blind spots in biosecurity practices in Estonia.

**Supplementary Information:**

The online version contains supplementary material available at 10.1186/s13028-025-00822-9.

## Background

African swine fever (ASF) has remained a persistent concern in Estonia since 2014, affecting both domestic pig and wild boar populations. Following a gap of over 3 years (2018–2020) without reported ASF outbreaks in domestic pigs, in July 2021, a pig farm reported an outbreak, marking the end of the prolonged absence of ASF in domestic pig populations. Subsequently, the latest ASF outbreaks in two domestic pig holdings from the same region were reported in 2023, during the summer period [1, 2]. Over the past two decades, the number and significance of the backyard sector and the numbers of smallholders have decreased considerably. Pig production has become highly industrialised and is predominantly conducted on commercial farms in Estonia [3]. In 2023, according to the national animal register, approximately 271,000 pigs were kept in Estonia across 129 pig farm units, which were owned by around 70 registered pig owners [4].

Presently, two primary mechanisms are considered responsible for the spread of ASF: local transmission facilitated by the migration of wild boar, and trans-regional human-mediated spread, occasionally spanning significant distances [5, 6]. In EU Member States, restricted zones I, II and III with special disease control measures are applied depending on the epidemiological situation of ASF and transmission risk [7]. Due to the ongoing epidemic in the wild boar population, the Republic of Estonia (apart from the island of Hiiumaa, where ASF has never been detected) is currently designated as restricted zone II.

Participatory methods were employed with hunters in Estonia to assess their perceptions of ASF control and surveillance in wild boars [8]. Additionally, another participatory study was conducted with pigkeepers to reveal their awareness, attitudes, and perceptions of ASF and the associated control measures [9]. By incorporating insights from these studies, there is a greater likelihood of implementing strategies embraced by the main stakeholders, enhancing the prospects for successful control of ASF in domestic pig and wild boar populations. However, the perceptions of veterinarians, another important player in ASF control and prevention, have remained unexplored in Estonia. The engagement of farm veterinarians is crucial given their critical role in offering guidance to pigkeepers, monitoring for signs of disease, collecting samples, and enforcing biosecurity measures on farms. Their perspectives will foster a collaborative and informed approach to managing ASF in the pig farming industry in the affected regions. Through these insights, specific interventions can be tailored to address knowledge gaps, identify initial steps for enhancing biosecurity practices on farms, and encourage collaboration among stakeholders. As a continuation of previous studies, the objective of this study was to explore the awareness of veterinary staff working on commercial pig farms regarding the signs, transmission routes, and preventive measures for ASF. In addition, we sought to assess their acceptance of ASF control measures and to identify obstacles encountered in implementing and maintaining biosecurity measures against ASF on farms. Finally, this study aimed to investigate the awareness of stakeholders involved in ASF control, the perception of their roles, and the level of trust in their capability to perform these roles.

## Methods

### Focus groups

The target group for this study consisted of veterinarians and veterinary assistants working in commercial pig farms in Estonia. Veterinary assistants do not hold a veterinary degree but have vocational education of veterinary technician or may have received on-the-job training from a veterinarian to perform specific tasks.

Participants were recruited using convenience sampling and were contacted via phone calls and personal connections. During the invitation process to join the focus group (FG), participants were informed about the study’s aims, methodology, and voluntary participation. For each meeting, the aim was to recruit three to five participants. The meetings took place between January and October 2023 in rented seminar rooms located near the participating farms, or at university facilities. All participants provided written consent for audio recording and anonymous data use. Focus group discussions (FGDs) were conducted in the Estonian language by a facilitator trained in the applied methodology and lasted for approximately two hours. Prior to the FGD, the facilitator asked participants about the characteristics of their clients’ farms, including the total number of pigs across those farms. To maintain anonymity, the participants’ names and demographic information were not asked. The gender and age of the participants were evaluated visually by the facilitator. Subsequently, audio recordings were translated into English by professional interpreters at the Language Centre of the Estonian University of Life Sciences. During the analysis of the translated transcripts, if it was evident that participants had experienced an ASF outbreak, this information was noted by the researcher.

### Tasks of FGD and employed tools

The meeting protocol consisted of three parts: introduction to the study and methodology, signing of a written consent form, and performing six specific tasks (Table 1). Tasks 1–3 focused on recognizing ASF and understanding the potential infection pathways in the herd as well as measures to prevent virus introduction. Task 4 addressed the obstacles encountered during the implementation and maintenance of biosecurity measures against ASF on pig farms. Task 5 explored the participants’ acceptance of various control measures. Lastly, Task 6 focused on stakeholders in ASF control, and the participants’ trust in these stakeholders to fulfil their respective roles. The employed tools were adapted from the previous studies [8, 10]. Proportional piling was employed to enable the entire group to determine the relative importance of the listed characteristics or statements (called ‘items’ hereafter), depending on the task. Participants were instructed to divide 100 glass beads into piles, proportional to their opinion for each listed item, without counting them. After the discussion group reached consensus, the facilitator counted and recorded the number of glass beads allocated to each listed item.

**Table 1:** Descriptions of focus group discussion topics and employed tools

Topic	Assignment	Assignment output and used tools
1. ASF^1^ signs in domestic pigs	1.1 Please name the first signs in the herd that would lead you to suspect ASF infection	Compilation of a list
2. ASF transmission routes	2.1. Please name the possible transmission routes of ASF virus to the pig farm	Compilation of a list
	2.2. Please assess which of the listed transmission routes pose the highest risk of introducing ASF virus into the herd in Estonia	Tool: proportional piling
3. ASF preventive measures	3.1. Please name the measures that can be used to prevent the introduction of ASF virus into the herd	Compilation of a list
	3.2. Please express which of these listed preventive measures you consider the most effective in preventing ASF introduction into the herd	Tool: proportional piling
4. Obstacles to the implementation and maintenance of ASF biosecurity measures	4.1. Please name the most important obstacles to the implementation and maintenance of biosecurity measures against ASF	Compilation of a list
	4.2. Which of these listed obstacles have the greatest impact?	Tool: proportional piling
5. ASF control measures	5.1. In the event of an ASF outbreak on a farm, the following control measures are implemented: • Culling of all animals on the farm • Destroying the feed and bedding materials on the farm • Farm cleaning and disinfection • Farm quarantine • Establishing a protection and a surveillance zone • Zoning (establishment of I, II, III restricted zones) Please express how acceptable you find each of these control measures	Tool: face emojis
6. Stakeholders involved in ASF control	6.1. Please name all the institutions and individuals involved in the development, implementation and surveillance of ASF prevention and control measures	Compilation of a list
	6.2. Please express your opinion on the importance (role) of each listed stakeholder in ASF prevention and control	Tool: Proportional piling
	6.3. Please express your level of trust in listed stakeholders regarding their ability to fulfil their role in ASF control	Tool: 10-beads score

^1^African swine fever

**Fig. 1:**
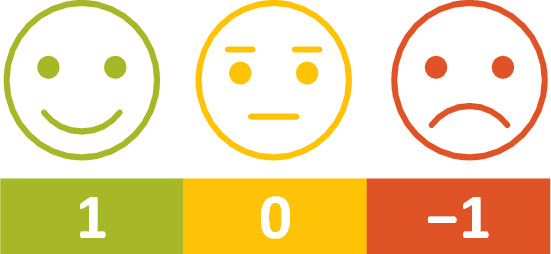
Visualisation tool illustrating acceptance of veterinary staff in Estonia (assigned to ranks from 1 to − 1)

Face emojis, designed in three colours, were used to capture the participants’ individual opinion. The emojis included a negative facial expression in red, a neutral facial expression in yellow, and a positive facial expression in green (Fig. 1).

Additionally, we introduced a 10-beads individual scoring tool (Table 1, Assignment 6.3), enabling participants to express their trust in the stakeholders’ ability to fulfil their respective roles in ASF control. Each participant received a set of ten glass beads and was instructed to use these beads to assign a score from zero (complete mistrust) to ten (complete trust) to each stakeholder listed on the paper.

In every task the facilitator promoted discussion by asking why participants provided certain answers. Qualitative findings from the recorded FGDs were descriptively incorporated into the analysis.

### Data management and analysis

After each FG meeting, the collected data were entered into Microsoft Excel (2019) spreadsheets and translated into English. The entire dataset in the Excel file was rechecked to avoid possible insertion mistakes. Items listed by different FGs using different wordings but conveying similar meanings were re-coded and grouped under common categories (see Additional file 1: Table S1). The term ‘listed item’ refers hereinafter to participant-generated items as well as to those that were predefined (enforced) by the research team.

For the analysis of the proportional piling tool (Table 1, Tasks 2, 3, 4, 6), the adjusted glass beads scores attributed to an item by each group were summed. The adjustment was based on how many items were listed by the group relative to the number of all unique items listed by all groups. The sum of the weighted proportional piling score ${S}_{i}$ for each listed item $i$ was calculated as follows:

1 $${S}_{i}={\sum }_{j=1}^{K}G{B}_{ij}\cdot \frac{{N}_{j}}{{N}_{total}}$$

, where $K$ is the total number of focus groups; $G{B}_{ij}$ is the number of glass beads assigned to the listed item $i$ by participants from group $j$; ${N}_{j}$ is the number of items listed by group $j$; ${N}_{total}$ is the number of all unique listed items in all groups. If a group $j$ did not list an item $i$ the value $G{B}_{ij}$ was assigned to zero.

For the analysis of face emojis (Table 1, Task 5), numerical values: + 1 for positive, 0 for neutral, and − 1 for negative expression (Fig. 1) were assigned to each response. Then numerical values given by all voting participants to the predefined listed item were summed. Finally, the sum was divided by the total number of voting participants $P$. The average emoji score ${A}_{i}$ for each listed item $i$ was calculated as follows:

2 $${A}_{i}=\frac{1}{P}{\sum }_{p=1}^{P}{E}_{ip}$$

$${E}_{ip}\in \{-\text{1,0},1\}$$

where ${E}_{ip}$ denotes one of the three face emojis assigned to the listed item $i$ by participant $p$ and assigned to the corresponding rank (Fig. 1).

For the analysis of the 10-bead individual scoring tool (Table 1, Task 6), the mean score value for each listed item was calculated by summing the scores given by participants from all focus groups in which it was listed. The sum was divided by the total number of participants in those focus groups that listed that item. The average of 10-bead individual score ${T}_{i}$ of each listed item $i$ was calculated as follows:

3 $${T}_{i}=\frac{{\sum }_{j=1}^{K}{\sum }_{p=1}^{{P}_{j}}{T}_{i,jp}}{{P}_{i}^{total}}$$

where $K$ is the total number of focus groups, ${T}_{i,jp}$ is an assigned trust score by participant $p$ from group $j$ regarding the listed item $i$; ${P}_{i}^{total}$ is the total number of participants among groups that listed item $i$; ${P}_{j}$ is the number of participants in group $j$; If a group $j$ did not list an item $i$ the value ${T}_{i,jp}$ was assigned to zero for all participants.

## Results

### Recruitment of participants

In total, five meetings were conducted with two to four participants per FG (Table 2). The study included 11 farm veterinary surgeons (6 female, 5 male) and 4 veterinary assistants (4 female). Participants’ estimated age ranged from 20 to 70 years, with approximately four aged under 40, six between 40 and 60, and four over 60. During the discussions, participants in the three FGs revealed that they had experienced a confirmed ASF outbreak.

**Table 2:** Demographic data of the participants and characteristics of their farms

Focus group	Gender	Approx. age	Farm characteristic	Occupation
1	Male	> 60	Fattening farm with 10,000 pigs	veterinarian
	Male	> 60	2 fattening farms with 7200 pigs in each	veterinarian
	Female	< 30	Breeding farm with 3800 sows	veterinary assistant
	Female	40–50	Breeding farm with 3800 sows	veterinary assistant
2	Female	< 30	Farrow to finish farm with 7000 pigs; Farrow to finish farm with 3000 pigs	veterinarian
	Female	< 30	Farrow to finish farm with 500–600 sows	veterinarian
	Female	40–50	Farrow to finish farm with 500 sows	veterinary assistant
3	Female	> 60	10 farms, different types, in total 30,000 pigs	veterinarian
	Female	40–50	Fattening farm with 3000 pigs; Farrow to finish farm with 1500 pigs	veterinary assistant
4	Female	30–40	Farrow to finish farm with 1000 pigs	veterinarian
	Female	40–50	18 farms, different types, in total 62,000 pigs	veterinarian
	Male	40–50	Farrow to finish farm with 2000 pigs	veterinarian
5	Female	> 60	Farrow to finish farm with 60,000 pigs	veterinarian
	Male	40–50	Fattening farm with 2000 pigs	veterinarian
	Male	50–60	7 farms, different types, in total 28,000 pigs	veterinarian

### Task 1. ASF signs in domestic pigs

Altogether, the veterinary staff from the five FGs listed 15 different signs indicative to ASF infection in the herd (Table 3). The most frequently mentioned sign was haemorrhages on skin, mucosa and organs (*n* = 5). This was followed by fever, loss of appetite, and increased mortality (*n* = 4). While participants mentioned increased mortality, their descriptions of it varied. During discussions, participants in the first FG highlighted increased mortality (several pigs dying in the same pen), developing over a couple of weeks after the first death was observed. The second group mentioned sudden deaths (*n* = 1) and high mortality numbers in certain parts of the pigsty (one pen or neighbouring pens). The third group specified increased mortality in one section of the pigsty. The fourth group emphasized increased mortality among the sows. The last group that did not list increased mortality revealed that they assumed mortality to be high, but it appeared to be limited to a few deaths within a single pen, which they did not consider indicative. In the case of no response to treatment (*n* = 2), participants revealed beginning suspecting ASF. All groups discussed postmortem signs and, in addition to haemorrhages, also listed spleen anomalies (*n* = 3), filled and darkened gallbladder (*n* = 1). One group highlighted the difficulty of describing a classical autopsy because of its potentially atypical nature. Participants from three groups (two groups had ASF experienced participants) emphasized that autopsies provided typical findings.

**Table 3:** First signs that would lead veterinary staff to suspect ASF in pigs

Sign	Groups listing (n)^a^
Haemorrhages on skin, mucosa and organs	5
Fever	4
Loss of appetite	4
Increased mortality	4
Spleen anomalies	3
No response to treatment	2
Nose bleeding	2
Lethargy	2
Non-clotting of the blood	2
Abortions, stillbirths	2
Increased morbidity	1
Sudden death	1
Bloody stool	1
Filled and darkened gull bladder	1
Detachment of the top skin layer	1

^a^Total number of focus groups was 5

### Task 2. ASF transmission routes

Among the 20 transmission routes listed (Table 4), people, transport vehicles, and bedding were designated the highest risk of introducing ASFV into the herd. The greatest risk was attributed to human-mediated transmission, with three groups broadly referring to people (score = 29.3). Additionally, two other groups specified subcategories: farm workers (score = 20.8), service providers (score = 10.4), and visitors (score = 7.8). In summary, discussions highlighted the risks associated with the failure of individuals to adhere to farm rules (changing clothes, shoes after forest activities, carrying personal phones), the role of humans in introducing viruses through vehicles, and deliberate acts. According to veterinarians, the tendency among farm workers to adhere less to biosecurity measures over time could be attributed to complacency and decreased vigilance as the perceived threat of ASF may seem distant. Additionally, the risk stemmed from farm visitors potentially deceiving staff about their compliance with farm rules, such as falsely claiming they had not visited forests or travelled abroad within the 48 h prior to arriving at the farm, or denying they had brought personal items like phones onto the premises. Veterinary staff disclosed that service providers presented a unique challenge owing to their essential role in farm operations, lack of control over their adherence to biosecurity measures, and the urgency of their services during emergencies. Unlike typical farm visitors, service providers could not be restricted for 48 h prior to visiting the farm and enforcing strict hygiene measures was challenging. Transport vehicles ranked second (*n* = 3, score = 27.7) due to frequent movement between different locations. Additionally, farm machinery, which was mentioned by two other groups, ranked fifth (score = 19.6) as highlighted due to their possible farm proximity during the fieldwork seasons. The involvement of humans in operating and transferring viruses from machinery to farms was mentioned. Bedding material ranked third (*n* = 5, score = 25.3), with straw being less common on farms and unable to be effectively heat-treated, like sawdust. In particular, if not stored for the required 90 days, straw was identified as a high-risk factor owing to the potential for contamination from wild boars visiting grain fields. Some participants noted that the use of bedding, required to meet higher animal welfare standards, may further elevate the risk of ASF transmission. Insects ranked sixth (*n* = 4, score = 18.8) and participants indicated uncertainty regarding their role in transmitting ASF to pig farms in Estonia.

**Table 4:** Transmission routes ranked by veterinary staff’s perceived risk of ASF introduction to the herd

Transmission route	Groups listing (n)^a^	Perceived risk (score^b^)
People	3	29.3
Transport vehicles	3	27.7
Bedding material	5	25.3
Farm workers	2	20.8
Farm machinery	2	19.6
Insects	4	18.8
Feed	3	16.7
Fresh fodder or forage without thermal treatment	2	14.5
Tools and equipment	3	14.2
Service providers	2	10.4
Infected pig	2	9.4
Wild animals	3	8.5
Visitors	2	7.8
Rodents	4	7.2
Cats and dogs	3	6.7
Food waste	2	5.1
Semen	1	3.3
Birds	2	2.8
Air	1	2.0
Heat treated forage	1	0.5

^a^Total number of focus groups was 5; ^b^Sum of the weighted proportional piling score

### Task 3. ASF preventive measures

Among the 30 listed preventive measures (Table 5), training of people, work instructions, biosecurity plan ranked first (*n* = 3, score = 20.7) in terms of effectiveness in preventing ASF introduction into herds. From the discussions, repeating detailed instructions and emphasizing the reasons behind the rules increased compliance and awareness. Additionally, using clear visual signs helped convey important information, especially when language barriers existed, or when workers were tired. Disinfection and washing of vehicles and people ranked second (*n* = 4, score = 19.2), with two groups commenting doubtful disinfection effectiveness during the cold seasons. Movement restrictions for vehicles and people ranked third (*n* = 3, score = 16.7). In the discussion, the prohibition of hunters from entering farms and the restriction of workers’ movement between different zones and animal groups within the farm was emphasized. Fencing was ranked fourth (*n* = 4, score = 15.9), as an important barrier against strangers and wild animals.

**Table 5:** Preventive measures ranked by veterinary staff’s perceived effectiveness in preventing ASF introduction into the herd

Preventive measure	Groups listing (n)^a^	Perceived effectiveness (score^b^)
Training of people, work instructions, biosecurity plan	3	20.7
Disinfection and washing of vehicles and people	4	19.2
Movement restrictions for vehicles and people	3	16.7
Fencing	4	15.9
Changing of clothes and footwear	3	13.2
Sauna and showering before entering pig facility	3	10.9
Heat treatment and withdrawal period for feed	4	9.2
Restricting access to visitors (applying 48 h pig free period)	3	9.1
Disinfection barrier for people	2	8.7
Avoiding use of litter or applying 90 days withdrawal period	3	6.2
Insect nets on ventilation openings, windows and doors	2	5.6
Disinfection of tools	2	4.9
Disinfection barrier for vehicles	1	4.7
Prohibition of bringing own food to the pig facility	2	4.4
Rodent, insect, bird control	2	4.3
Change of clothes, shoes + washing	1	4.3
Logistics (avoidance of cross-contamination)	1	3.7
Waiting period for tools 24 h (quarantine storage)	1	3.6
ASF biosecurity plan	1	3.3
Protective clothing for service providers	1	2.8
Changing footwear	1	2.7
Registration of entering people	2	2.6
Clean and dirty zones within the pighouse	1	2.1
Rodent control	1	2.0
Planning of carcass transport	1	1.9
Personal protective equipment	1	1.1
Keeping in order the territory of the farm	1	1.1
Traps for cats	1	0.9
Checking the health status of the farm of origin of pigs	1	0.5
Fly control	1	0.3

^a^Total number of focus groups was 5; ^b^Sum of the weighted proportional piling score

### Task 4. Obstacles to the implementation and maintenance of ASF biosecurity measures

Among the 16 obstacles listed (Table 6), the motivation and attitudes of staff (*n* = 5, score = 44.0) and financial constraints (*n* = 5, score = 41.8) had the greatest perceived impact on the implementation and maintenance of ASF biosecurity measures. From the discussions, the major obstacles were the mindset and attitudes of farm employees, including sometimes the farm managers. Participants indicated that workers may exhibit negligence and carelessness because of their perception that problems were not their responsibility. This behaviour was also attributed to burnout and a false sense of security stemming from a prolonged period without disease outbreaks. A lack of knowledge and understanding among workers about the risks involved in non-compliance with biosecurity measures was also discussed. In terms of financial constraints, discussions from all groups highlighted the significant impact of extra biosecurity costs (for disinfectants, clothing, etc.) on the farm economy. Often, farms had to make tough choices due to limited resources, prioritising immediate needs like feeding animals, over other expenses. Even small costs added up, creating financial constrain and leading to compromises on biosecurity measures. Participants revealed that many farms could not afford to build essential infrastructure like disinfection baths, relying instead on cheaper alternatives, such as regular sprays. Smaller farms were particularly affected. As highlighted, there was a general lack of compensatory measures for the costs incurred in biosecurity efforts. Farmers felt abandoned with insufficient support from the state, who conducted checks but did not offer substantial aid. Additionally, several factors were listed as obstacles to preventing or controlling ASF in the country in general, such as the large wild boar population (*n* = 1, score = 9.0), poor communication with hunters (*n* = 1, score = 6.4), lack of a vaccine (*n* = 1, score = 5.6) and open border with Russia for wild boar movements (*n* = 1, score = 5.3).

**Table 6:** Obstacles ranked by veterinary staff’s perceived impact on implementing and maintaining ASF biosecurity measures

Obstacles	Groups listing (n)^a^	Perceived impact (score^b^)
Motivation and attitudes of farm employees	5	44.0
Financial constraints	5	41.8
Shortage, quality and cost of labour	2	12.3
Large wild boar population	1	9.0
Weather conditions	2	8.4
Non-optimal farm management (planning)	1	7.0
Involvement of the farm executives	1	6.6
Poor communication with hunters	1	6.4
Force majeure (e.g. power cuts, etc.)	2	6.1
Lack of vaccine	1	5.6
Open for wild boar movement border with Russia	1	5.3
Legislation and inspections (welfare standards, disinfection in winter)	1	5.0
Involvement of partners (e.g. service providers)	1	4.8
Difficulty in deterring insects, birds	1	3.8
Delivery difficulties of supplies (e.g. disinfectants)	1	2.0
Extreme situations (emergencies, etc.)	1	0.9

^a^Total number of focus groups was 5; ^b^Sum of the weighted proportional piling score

### Task 5. ASF control measures

All participants accepted farm quarantine and cleaning and disinfection of the farm, recognising their role in eradicating the virus from infected farms (Table 7). The majority also accepted destroying feed and bedding materials on the farm, considering this a justified measure. At the same time, some participants discussed the possibility of testing feed before destruction, and the necessity of destroying it depending on storage conditions and duration. Others have discussed the economic feasibility of destroying versus preserving and alternative uses of feed. Establishing protection and surveillance zones was accepted by most participants. Some also raised concerns about the implementation principles, such as the size of the zones and the duration of restrictions. It was highlighted that the implementation often lacked consideration of local geography, leading to arbitrary zone boundaries that may not be practical, causing undue hardship and confusion. Culling of all animals on the farm was not universally accepted. The majority acknowledged the necessity of culling, as there were no viable alternatives, and infected animals posed a significant risk of spreading the disease. As discussed, while some animals may not be immediately sick, the risk of missing an infected one necessitates culling all to prevent the disease from persisting. Additionally, some emphasised the decision’s inevitability due to the existing legal framework and the absence of any treatment. Some participants also advocated a case-by-case approach, considering factors such as the extent of infection and farm layout. Three groups expressed the desire to find humane and practical ways to use the animals, rather than just wasting them, while acknowledging the challenges and risks involved. One group suggested using the animals for food, as was done according to their knowledge in countries like Bulgaria, Romania, and Russia, while another group suggested heat-treating the meat or using it in dog food or other products. According to participants, culling is emotionally challenging, particularly when animals are healthy or when only a small number of animals are affected. Finally, the establishment of restricted zones I, II, and III received the least acceptance, with discussions focusing on their adverse effects on market access, livestock product prices, and economic hardships for farmers. Some criticised inconsistencies in zoning practices, including variations in interpretations and implementations across EU Member States.

**Table 7:** African swine fever control measures ranked by veterinary staff’s acceptance

Control measure	Acceptance (score^a,b^)
Farm quarantine	1
Farm cleaning and disinfection	1
Destroying the feed and bedding materials on the farm	0.7
Establishing protection and surveillance zones	0.6
Culling of all animals on the farm	0.1
Zoning (establishment of I, II, III restricted zones)	− 0.6

^a^Total number of participants was 15; ^b^Average emoji score

### Task. 6 Stakeholders involved in ASF control

Altogether, 26 stakeholders who contributed to the development, implementation and surveillance of ASF control and prevention measures in Estonia were listed (Table 8). Pigkeepers were assigned the highest importance (*n* = 5, score = 34.5) to their role in implementing biosecurity measures to prevent disease outbreaks. From the discussions, their role was to ensure compliance with biosecurity regulations, including providing the necessary equipment and supplies. Farm owners are expected to implement both mandatory and voluntary biosecurity measures, and some participants believed that owners should go beyond the minimum requirements. In general, the veterinary staff expressed high levels of trust in the ability of pigkeepers’ to fulfil their role. Most participants noted that the majority of owners were committed to preventing disease outbreaks and ensuring biosecurity. At the same time, some had witnessed cases of negligence and non-compliance, which lead to mixed levels of trust. The Veterinary authority ranked second (*n* = 5, score = 21.2) and, as discussed, their role was policy enforcement, monitoring and surveillance efforts, and advisory support. Trust in the Veterinary authority varied widely as some trusted local officials more than the central office, citing individual competence and dedication. Others have noted low trust due to negative experiences and perceived inefficiencies. Issues such as a lack of expertise, insufficient administrative capacity, and staffing shortages were highlighted. Farm veterinarians ranked third (*n* = 2, score = 12.5) in terms of the importance of their role, which included checking and monitoring the fulfilment of biosecurity requirements on farms, diagnosing diseases, facilitating information exchange, and cooperation with pigkeepers. They had the highest level of trust in their ability to fulfil these roles. The Ministry of Rural Affairs ranked fourth (*n* = 3, score = 11.2) in terms of the role of developing legislation, decision-making, monitoring, advising, and informing farmers. In discussions concerning trust, participants highlighted a lack of support measures for pig breeders, current minimal dissemination of information about ASF, adoption of legislation without consulting stakeholders, and gaps in communication between the Ministry and the Veterinary authority. Farm workers ranked fifth (*n* = 2, score = 9.6) with the responsibility of ensuring compliance with biosecurity rules on farms. In general, participants expressed trust in farm workers in Estonia, with some noting certain workers’ positive attitudes and proactive approaches. Trust levels varied depending on the farm, as some workers required constant reminder and supervision. Laboratories ranked sixth (*n* = 3, score = 9.5) with the role of testing and diagnosing ASF. The discussion revealed varying trust levels in local laboratories. While some participants expressed confidence in their capabilities, others were sceptical due to what they perceived as past mistakes and concerns that outdated methods were still in use.

**Table 8:** Stakeholders in ASF control ranked by perceived important role and trust of veterinary staff

Trust (score^a^)	Stakeholder	Groups listing (n)^b^	Perceived role (score^c^)
7.8	Pigkeepers	5	34.5
5.7	Agriculture and food board (veterinary authority)	5	21.2
9.4	Farm veterinarians	2	12.5
3.8	Ministry of Rural Affairs	3	11.2
6.0	Farm workers	2	9.6
7.0	Laboratories	3	9.5
3.7	Hunters	1	9.3
7.3	Animal-waste processing plant	2	5.7
7.7	EU institutions	1	5.5
8.0	Slaughterhouses	1	5.5
4.1	Agricultural registers and information Board	2	5.4
7.4	Breeding association	2	5.1
4.0	Input vendors, suppliers	1	3.8
4.0	Drivers	1	3.7
7.3	Feed mills and manufacturers	1	3.4
4.6	Environmental board	2	3.0
5.0	Scientists	1	3.0
3.7	Transport companies	1	3.0
3.0	Government	1	3.0
3.0	Ministry of Environment	1	2.5
5.0	Bank	1	2.4
7.7	Estonian university of life sciences	1	1.8
6.3	Educational institutions (veterinary, animal husbandry)	1	1.7
5.0	Neighbouring farmers and landowners	1	1.3
3.8	Media and the general public	1	1.1
8.0	Police	1	0.5

^a^Average of 10-bead individual score; ^b^Total number of focus groups was 5; ^c^Sum of the weighted proportional piling score

## Discussion

This study targeted veterinary staff working on commercial pig farms under the assumption that their awareness, attitudes, and perceptions had the most direct impact on the implementation of disease control measures on farms. Our findings reflect the perspectives and opinions of veterinary staff working on commercial pig farms with approximately a decade of experience in dealing with ASF in an affected country.

Regarding ASF infection recognition in the herd, veterinary staff demonstrated a high awareness of the first early disease signs in the herd. The majority mentioned non-specific ASF signs as indicators of ASF in pigs, which aligns with a study of ASF outbreaks in Estonia, where the initial signs in herds were mild and non-specific [3]. Although each group mentioned at least one postmortem finding, some participants did not provide many details of the autopsies, which could be attributed to the framing of the question, focusing primarily on the initial early signs of ASF infection. Additionally, the majority indicated that high mortality is expected in certain part of the pig keeping facility (pen or section) rather than across the entire herd, demonstrating an understanding of the characteristics of disease spread within a pig herd [3].

Indirect transmission routes, such as by people, transport vehicles, farm machinery, and bedding materials were considered the most likely routes of virus introduction into Estonian pig herds, aligning with the perspectives of pigkeepers in Estonia [9]. The perceived relatively low risk of ASF introduction via direct transmission routes, such as direct contact with wild boars or introduction of infected pigs to the herd, could be explained by pig farming practices in Estonia. These practices effectively minimize the direct contact between domestic pigs and wild boars. In Estonia, pig production is highly industrialised and primarily concentrated on large farms. Outdoor pig keeping is prohibited, and all farms are enclosed by perimeter fences to prevent wild boars from accessing the farm territory [11]. Furthermore, animal movement is well controlled, and large pig farms do not introduce pigs from ‘unknown’ sources, thereby making the introduction of ASF with live animals less likely. Previous experiences with ASF outbreaks in domestic pigs in Estonia have also demonstrated that the most likely route of virus introduction has been of indirect nature [3]. The role of blood-sucking insects in ASFV transmission remains questionable, despite new evidence supporting their potential involvement [12]. Furthermore, the occurrence of ASF in domestic pigs in Estonia have shown a strictly seasonal pattern, coinciding with the peak activity of these insects [3]. This experience may have influenced the perception among veterinarians and pigkeepers [9] that the risk of virus introduction via blood-sucking insects is relatively high. The use of food waste for feeding pigs is illegal in Estonia and not practiced on large-scale farms. Furthermore, on most farms, staff are not allowed to bring their own food into pig facilities, and lunch is provided by owners to minimize the risk of introducing ASFV through accidental feeding of food scraps to pigs. Although food waste could potentially be fed to pigs on smallholder farms (although illegally), the number of such farms is very small in Estonia. This may have influenced veterinarians’ perception of the risk associated with introducing food waste (swill feeding), which is another globally recognized significant ASF transmission route [13, 14]. In general, a common understanding of ASF introduction risks among veterinarians and pigkeepers should create a good basis for collaboration in implementing preventive measures to mitigate these risks.

Certain tendencies emerged in the responses, indicating that the most effective preventive measures were related to mitigating the risks posed by the indirect transmission routes. Besides cleaning and disinfection barriers for people and vehicles, and fencing farm territory, heat treatment or applying withdrawal period for feed were frequently mentioned among the highly ranked preventive measures. According to recent research and risk assessments, cereal feeds have not been found to pose a high risk of ASFV introduction into farms [15]. However, feed (primarily fresh fodder) has been suspected as one of the plausible modes of introduction in several outbreak investigations in Estonia and Latvia [3, 16]. Furthermore, a withdrawal period for freshly harvested feed has been required by the national legislation for ASF control since 2015 [17]. These circumstances may have influenced the veterinarians’ opinions. As observed in our previous study with pigkeepers in Estonia [9], veterinarians likewise placed high importance on staff training and adherence to work instructions related to ASF preventive measures. This indicates a common understanding among veterinarians and pig farm managers regarding the importance of compliance in implementing these measures. In general, all compulsory biosecurity measures required by national legislation [18] were listed, and most were ranked as effective. This indicates that veterinarians not only know the legal requirements but also trust that they are meaningful. Measures such as insect nets on openings of pig facilities, use of sauna and showering before entering pig houses were considered effective, despite not being required by legislation. This demonstrates the readiness to implement biosecurity measures even stricter than those officially required.

Two major obstacles to the implementation and maintenance of biosecurity measures were highlighted: (1) the motivation and attitudes of farm employees and (2) financial constraints. Additionally, the two FGs emphasised shortage, quality, and cost of labour, which is closely related to the first two obstacles. As most commercial pig farms in Estonia use paid labour, including farm managers, they rely on employees’ motivation to fulfil the requirements established on the farm. The discussions revealed that veterinarians constantly face challenges with farm employees’ attitudes, and that it was often difficult to achieve a common understanding of the reasons for certain requirements between veterinarians and employees. Previous studies have demonstrated that a lack of knowledge and negative attitudes toward biosecurity hinder farmers’ implementation behaviours [19, 20]. Thus, all efforts of veterinarians and farm management staff that improve farm workers’ knowledge or shape their attitudes are as important as setting up disinfection barriers, hygiene locks, and operational protocols. Owing to the low profitability of the pig farming sector in Estonia over the last decade, the extra costs resulting from the implementation of biosecurity measures have had a significant economic impact on the pig farming sector. Consequently, several participants emphasised the need for financial support from the government for the implementation of costly biosecurity measures on pig farms. Examples include situations where the farmer had to choose between the basic needs of the pigs (feed) and biosecurity measures (disinfection) due to limited financial resources. To avoid this, additional support is needed. The remaining obstacles varied significantly between groups, with some factors considered general obstacles for the eradication or control of ASF. The diverse list of named obstacles likely reflected various problems that veterinarians have experienced in implementing these measures over the years. As it appears, these challenges seem to differ among veterinarians and, therefore, may not be so general. However, they provide an understanding of how complex the whole system is and how many little details may play a role in success or failure in preventing disease introduction.

The acceptability of compulsory disease control measures by stakeholders is important to ensure a good collaboration between the authorities and persons involved or affected by these measures, which in turn facilitates the proper implementation of the measures [21]. Farm quarantine, cleaning and disinfection were unanimously accepted by participants and did not raise any discussion. The destruction of feed and bedding materials present on the outbreak farm was not fully accepted by all participants, suggesting that depending on the storage conditions, safely stored feed could be saved and used for feeding other animal species. Culling all animals on the outbreak farm created the most discussions among participants. This is an example in which the necessity of the measure was rationally acknowledged by most participants but was emotionally not accepted. The most problematic for participants was the killing of healthy animals in large herds, where the disease had been detected in only a few animals within a single unit. A case-by-case approach was suggested considering the farm’s layout and the spread of infection within the farm, suggesting that pigs in unaffected farm units could be saved. In addition, alternative uses of culled animals have been proposed. The establishment of protection and surveillance zones around outbreak farms also raised variable opinions. Most participants accepted the measure, but some expressed concerns regarding the size and duration of restriction zones and the implemented restriction measures. However, there appeared to be some confusion among the participants regarding the different zone types. The restrictions imposed on farms located near the outbreak farm (protection and surveillance zone) were confused with the restrictions of the restricted zones I, II, and III which were discussed later in the task. Improved communication and education about these zones are necessary for farmers and farm veterinarians to ensure a better understanding and acceptance of these measures. The establishment of restricted zones I, II, and III in an affected country [7] appeared to be the least accepted, which was somewhat unexpected, as we assumed that veterinary staff would be better informed about the principles of disease control on a wider scale, including both the national and European Union levels. The adverse effects of zoning on pork product prices and the resulting economic hardships for farmers located in restriction zones were discussed. In this regard, the arguments coincided very much with those expressed by Estonian pig farm managers in our previous study [9]. This could be explained by the fact that most of the participating pig veterinarians provided services to pig farms of one owner or were employed by a single farm. In this way, they were aware of the farmers’ economic situation and were interested in their economic success, which might have shaped their opinions.

Knowing partners and their roles and responsibilities is a key precondition for successful collaboration in any activity, as it is for disease control and eradication. The lists of stakeholders compiled by each FG in our study were largely variable, with only two stakeholders named by all FGs: the pigkeepers and the Veterinary authority. This demonstrates that farm veterinarians recognise the crucial role of pigkeepers in preventing disease introduction to farms, and the role of Veterinary authority in implementing preventive measures and responding to outbreaks. However, the variable responses suggest that, in general, there is a lack of common understanding among farm veterinarians regarding other important parties involved in ASF control beyond those directly linked to disease control on farms. Interestingly, only one group recognised the role of hunters, who play a crucial role in ASF control in the wild boar population [8]. Some groups named the Environmental Board and the Ministry of Environment as stakeholders partly responsible for ASF control in wild boars, indicating that they consider this an important part of disease control in the country. Only two groups listed the farm veterinarians themselves as stakeholders in disease control. This may have been because of how the participants interpreted the question. As the facilitator did not provide instructions on how to interpret the question, participants might have assumed it referred to ‘other stakeholders’ besides themselves. For effective collaboration, there must be trust between the parties. Thus, we asked the participants to express their trust in the listed stakeholders in their ability to fulfil their roles. The two groups that named farm veterinarians as stakeholders, expressed the highest level of trust in themselves. Based on this, we could conclude that farm veterinarians are committed to their work and implementation of disease control measures or, at least, they highly value this commitment. The participants also expressed a high level of trust to pigkeepers despite bringing up some shortcomings during the discussions. Regarding governmental institutions, participants had the highest trust in the Veterinary authority, although the score was moderate compared to the highest scores given to other, more trusted stakeholders. It appeared that veterinarians tend to trust local veterinary officers more than the entire system. This could indicate good personal relationships between (at least some) officials working in the field and farm veterinarians, which is crucial for the success of common actions. Our results demonstrate that there is still room for improvement in communication across all stakeholder groups, which could increase the understanding, trust and collaboration between the parties involved.

Our findings suggest a need for more coordinated communication of disease control policies to stakeholders directly involved in it, to strengthen their understanding of the rationale behind current ASF control measures. This could be achieved through targeted training programs for veterinary staff and pigkeepers, and by facilitating discussion between veterinary authorities, pigkeepers, and pig-veterinarians. These discussions are planned as a next step, either integrated into regular meetings between pigkeepers and veterinarians or organized as dedicated sessions focused on these questions.

The representativeness of the study may be questioned due to the inclusion of only 15 participants; nonetheless, saturation of responses (when no new information is obtained) was achieved. There is a limited number of veterinarians working on pig farms in Estonia (about 20 veterinarians in total), and our sample included the majority of them working in this field. Additionally, most of them have long experience and stable positions, indicating limited professional turnover in Estonia. The exact number of veterinary assistants is unknown; however, they do not work on every farm in Estonia. All assistants working with recruited veterinarians were invited to participate and represent those working in farms serviced by the participating veterinarians. Owing to the nature of group dialogue, focus group discussions engage participants to interact and discuss, generating unique data distinct from individual interviews [22]. The influence of aspects like plurality and power dynamics could shape how individual views contrasted with the dominant perspective were expressed within the focus group [23]. To prevent dominant behaviour among participants, including those with ASF experience, a skilled facilitator ensured that everyone was heard and had equal opportunities to contribute. In this regard, all statements were considered and analysed, especially in tasks involving a group consensus answer. Considering the risk of information loss when translating recordings and dataset from Estonian to English [23], native-speaking research team members were consulted.

## Conclusions

- Veterinary staff working on Estonian pig farms demonstrated good awareness of ASF signs, transmission routes, and preventive measures.
- According to the veterinarians, the opinions, attitudes and mindsets of farm employees were perceived to play a pivotal role in the effective implementation of ASF preventive and biosecurity measures on pig farms.
- The herd-level ASF control measures were well accepted, except for the culling of all animals on the affected farm.
- In general, zoning, as an ASF control measure has been recognized as necessary to restrict disease spread. However, there are significant concerns about the fairness, consistency, and economic impact of these restrictions on farms, particularly regarding long term ASF restricted zones II, and III.
- Veterinary staff have an incomplete understanding of stakeholders in ASF control.
- Trust in Veterinary authorities was moderate, whereas trust in other government institutions remained low.
- The need for further training of veterinary staff in ASF control measures remains relevant, particularly in areas such as related EU and national legislation, involved parties and their roles, to ensure the effective implementation of measures and good collaboration with governmental institutions and other stakeholders.

## Supplementary Information

Additional file 1: Table 1. The table presents items listed by Estonian veterinary staff during Tasks 1–4 and 6, re-coded and grouped under common categories.

## Data Availability

The datasets used and analysed during the current study are available from the corresponding author upon reasonable request.

## Abbreviations

ASF: African swine fever
ASFV: African swine fever virus
FG: Focus group
FGD: Focus group discussion

## Declarations

### Prior publication

Data have not been published previously.

## References

[1] Ståhl K, Boklund AE, Podgórski T, Vergne T, Abrahantes JC, Cattaneo E, et al. Epidemiological analysis of African swine fever in the European Union during 2023. EFSA J. 2024;22:e8809. 10.2903/j.efsa.2024.8809. 38756349 10.2903/j.efsa.2024.8809 PMC11096997

[2] ADIS.: European Union Animal Diseases Information System. https://ec.europa.eu/food/animals/animal-diseases/animal-disease-information-system-adis_en. Accessed 5 April 2024.

[3] Nurmoja I, Mõtus K, Kristian M, Niine T, Schulz K, Depner K, et al. Epidemiological analysis of the 2015–2017 African swine fever outbreaks in Estonia. Prev Vet Med. 2020;181:104556. 10.1016/j.prevetmed.2018.10.001. 30482617 10.1016/j.prevetmed.2018.10.001

[4] Agricultural Registers and Information Board. https://www.pria.ee/. Accessed 16 May 2025.

[5] Boklund A, Cay B, Depner K, Földi Z, Guberti V, Masiulis M, et al. Epidemiological analyses of African swine fever in the European Union (November 2017 until November 2018). EFSA J. 2018;16:e05494. 10.2903/j.efsa.2018.5494. 32625771 10.2903/j.efsa.2018.5494 PMC7009685

[6] Chenais E, Depner K, Guberti V, Dietze K, Viltrop A, Ståhl K. Epidemiological considerations on African swine fever in Europe 2014–2018. Porc Health Manag. 2019. 10.1186/s40813-018-0109-2. 10.1186/s40813-018-0109-2 PMC6325717 30637117

[7] 2023/594/EU.: Commission Implementing Regulation (EU) 2023/594 of 16 March 2023. Laying down special disease control measures for African swine fever and repealing Implementing Regulation (EU) 2021/605. Off. J. Eur. Union, 2023. https://eur-lex.europa.eu/legal-content/EN/TXT/?uri=CELEX%3A32023R0594. Accessed 5 April 2024.

[8] Urner N, Mõtus K, Nurmoja I, Schulz J, Sauter-Louis C, Staubach C, et al. Hunters’ acceptance of measures against African swine fever in wild boar in Estonia. Prev Vet Med. 2020;182:105121. 10.1016/j.prevetmed.2020.105121. 32818692 10.1016/j.prevetmed.2020.105121

[9] Moskalenko L, Schulz K, Mõtus K, Viltrop A. Pigkeepers’ knowledge and perceptions regarding African swine fever and the control measures in Estonia. Prev Vet Med. 2022;208:105717. 10.1016/j.prevetmed.2022.105717. 35985184 10.1016/j.prevetmed.2022.105717

[10] Moskalenko L, Schulz K, Nedosekov V, Mõtus K, Viltrop A. Understanding smallholder pigkeepers’ awareness and perceptions of African swine fever and its control measures in Ukraine. Pathogens. 2024. 10.3390/pathogens13020139. 38392877 10.3390/pathogens13020139 PMC10893472

[11] European Food Safety Authority (EFSA), Nielsen SS, Alvarez J, Bicout DJ, Calistri P, Canali E, Drewe JA, et al. Scientific opinion on the African swine fever and outdoor farming of pigs. EFSA J. 2021;19:6639. 10.2903/j.efsa.2021.6639. 10.2903/j.efsa.2021.6639 PMC8188572 34140998

[12] Olesen AS, Stelder JJ, Tjørnehøj K, Johnston CM, Lohse L, Kjær LJ, et al. Detection of African swine fever virus and blood meals of porcine origin in hematophagous insects collected adjacent to a high-biosecurity pig farm in Lithuania; a smoking gun? Viruses. 2023. 10.3390/v15061255. 37376554 10.3390/v15061255 PMC10301464

[13] Bellini S, Casadei G, Lorenzi GD, Tamba M. A review of risk factors of African swine fever incursion in pig farming within the European Union scenario. Pathogens. 2021;10:84. 10.3390/pathogens10010084. 33478169 10.3390/pathogens10010084 PMC7835761

[14] Dame-Korevaar A, Boumans IJMM, Antonis AFG, van Klink E, de Olde EM. Microbial health hazards of recycling food waste as animal feed. Future Foods. 2021;4:100062. 10.1016/j.fufo.2021.100062.

[15] Blome S, Schäfer M, Ishchenko L, Müller C, Fischer M, Carrau T, et al. Survival of African swine fever virus in feed, bedding materials and mechanical vectors and their potential role in virus transmission. EFSA Support Publ. 2024;21:8776E. 10.2903/sp.efsa.2024.EN-8776.

[16] Oļševskis E, Guberti V, Seržants M, Westergaard J, Gallardo C, Rodze I, et al. African swine fever virus introduction into the EU in 2014: experience of Latvia. Res Vet Sci. 2016;105:28–30. 10.1016/j.rvsc.2016.01.006. 27033903 10.1016/j.rvsc.2016.01.006

[17] Ministry of Rural Affairs.: Põllumajandusministri 23. novembri 2004. a määruse nr 179 “Sigade klassikalise katku ja sigade aafrika katku tõrje eeskiri” muutmine. Vastu võetud 06.08.2015 nr 78. https://www.riigiteataja.ee/akt/107082015019. Accessed 1 June 2023.

[18] Regulation of Ministry of Agriculture.: Sigade klassikalise katku ja sigade Aafrika katku tõrje eeskiri. Vastu võetud 23.11.2004 nr 179. https://www.riigiteataja.ee/akt/821454. Accessed 1 June 2023.

[19] Alarcon P, Wieland B, Mateus ALP, Dewberry C. Pig farmers’ perceptions, attitudes, influences and management of information in the decision-making process for disease control. Prev Vet Med. 2014;116:223–42. 10.1016/j.prevetmed.2013.08.004. 24016600 10.1016/j.prevetmed.2013.08.004

[20] Ellis-Iversen J, Cook AJC, Watson E, Nielen M, Larkin L, Wooldridge M, et al. Perceptions, circumstances and motivators that influence implementation of zoonotic control programs on cattle farms. Prev Vet Med. 2010. 10.1016/j.prevetmed.2009.11.005. 19963291 10.1016/j.prevetmed.2009.11.005

[21] Gisclard M, Charrier F, Trabucco B, Casabianca F. From national biosecurity measures to territorial ASF preparedness: the case of free-range pig farming in Corsica. France Front Vet Sci. 2021;8:689163. 10.3389/fvets.2021.689163. 34395574 10.3389/fvets.2021.689163 PMC8355427

[22] Krueger RA. Focus groups: a practical guide for applied research. Washington: SAGE Publications; 2014. p. 1–280.

[23] Fischer K, Schulz K, Chenais E. Can we agree on that? Plurality, power and language in participatory research. Prev Vet Med. 2020;180:104991. 10.1016/j.prevetmed.2020.104991. 32422475 10.1016/j.prevetmed.2020.104991

