# Supplementary material for: Veterinarians’ perceptions on African swine fever and the control measures in Estonia
Source: Acta Vet Scand. 2025 Aug 27;67:41. doi: 10.1186/s13028-025-00822-9 (PMC12382203; doi:10.1186/s13028-025-00822-9)
Supplement: Supplementary file 1 — Additional file 1: Table 1. The table presents items listed by Estonian veterinary staff during Tasks 1–4 and 6, re-coded and grouped under common categories. [file 13028_2025_822_MOESM1_ESM.docx]

Supplementary table S1. The table presents items listed by Estonian veterinary staff during Tasks 1–4 and 6, re-coded and grouped under common categories.

| Task | Common notation | Listed item by the focus group |
| --- | --- | --- |
| 1. African swine fever signs in domestic pigs | Loss of appetite | Loss of appetite |
|  |  | Decreased appetite |
|  |  | Loss of appetite |
|  |  | Loss of appetite |
|  | Haemorrhages on skin, mucosa and organs | Vascular permeability (hemorrahages, bruising) |
|  |  | Hemorrhages on the body, pettechiae (on organs, mucuous membranes) |
|  |  | Haemorrhagies in organs, Pink skin color - haemorrhages |
|  |  | Pettechiae on parenchymal organs |
|  |  | Haemorrhages on the mucous membranes, organs, on the skin |
|  | Spleen anomalies | Anomaly of spleen |
|  |  | Spleen changed (soft, lumpy, enlarged, dark) |
|  |  | Spleen slightly enlarged |
|  | Increased mortality | Increased mortality (in a short period of time) |
|  |  | Increased mortality (starts from one location). |
|  |  | Increased mortality |
|  |  | Increased mortality |
|  | Nose bleeding | Non-clotting of the blood (nosebleeds) |
|  |  | Bloody discharge from the nose |
|  | Lethargy | Lethargic |
|  |  | Lethargy |
|  | Non-clotting of the blood | Bleeding of injection sites |
|  |  | Non-clotting of the blood |
|  | Abortions, stillbirths | Abortions, stillbirths |
|  |  | Abortion |
| 2. African swine fever transmission routes | Infected pig | By another pig |
|  |  | Live animals (pigs) |
|  | Transport vehicles | Transportation vehicles |
|  |  | Transportation vehicles (serving) |
|  | Fresh fodder or forage without thermal treatment | Green fodder, forage without thermal treatment |
|  |  | With feed (unheated) |
|  | Service staff | Service staff |
|  |  | Service providers |
|  | Farm workers | Farm worker (seasonal risks) |
|  |  | Workers |
|  | Visitors | Visitors (personal items) |
|  |  | Visitors (people) |
|  | Wild animlas | Wild animals |
|  |  | Wild animals |
|  |  | Game animals (fox, racoondog, etc.) |
|  | Bedding material | Bedding |
|  |  | Bedding (deep) |
|  |  | With bedding (straw) |
|  | Insects | Blood sucking insects |
|  |  | Flies |
|  |  | Insects |
|  | Tools and equipment | Tools and consumable (incl. medicines, tools, telefonid etc.) |
|  |  | Inventory |
|  |  | Goods, accessories |
|  | Cats and dogs | Domestic animals (dogs) |
|  |  | Domestic animals |
|  |  | Cat |
|  | Food waste | Human food |
|  |  | Food taken to the farm |
| 3. African swine fever preventive measures | Disinfection and washing of vehicles and people | Disinfection and washing (vehicles and people) |
|  |  | Desobarriers (technique + people) |
|  |  | Desovans / mats |
|  |  | Deso baths / mats / house |
|  | Changing of clothes and footwear | Changing of clothes and footwear |
|  |  | Change of clothes, shoes |
|  |  | Change of clothes |
|  | Movement restricitions for vehicles and people | Movement restriction (incl. barriers like fencing, machinery, people) |
|  |  | Restriction of movement (people, transport) |
|  |  | Separation of zones |
|  | Prohibition of bringing own food to pig facility | Forbid employees to take lunch to the farm (no leftovers to pigs) |
|  |  | Organized catering (without pork) |
|  | Heat treatment and withdrawal period for feed | Fodder |
|  |  | Withdrawal period for freshly harvested crops (2-3 month) |
|  |  | Feed standing time, storage conditions |
|  |  | Heat treatment of feed |
|  | Avoiding use of litter or applying 90 days withdrawal period | Applying 90 days wating time after harvest to bedding material |
|  |  | Storage of bedding (90days) |
|  |  | Standing time of the litter, storage conditions |
|  | Registration of entering people | Registration of entering people |
|  |  | Visits registration check - on paper |
|  | Restricting access of visitors (applying 48 h pig free period) | Limiting visitors (48 hour waiting period rule) |
|  |  | Restriction of strangers |
|  |  | Standard procedure for entering the zone (washing, sauna, protective clothing) |
|  | Sauna and showering before entering pig facility | Washing (people), sauna |
|  |  | Washing, sauna |
|  | Disinfection barrier for people | Desobarriers for people (mats, hand deso, protective clothing) |
|  |  | Shoe and hand deso at the gate |
|  | Insect nets on ventilation openings, windows and dors | Nets on windows and doors |
|  |  | Nets on windows and on ventilation openings |
|  | Training of people, work instructions, biosecurity plan | Training of people (labels, instructions), work instructions, biosafety plans |
|  |  | Notification of people |
|  |  | Explanatory work |
|  | Disinfection of tools | Deso of inventory |
|  |  | Deso of working tools |
| 4. Obstacles to the implementation and maintenance of African swine fever biosecurity measures | Shortage and quality of labour, labour costs | Shortage of labour, costs, quality |
|  |  | Lack of skilled workers |
|  | Finanacial constraints | Additional cost (disinfectants, clothing, equipment, etc.) |
|  |  | Financial constraints |
|  |  | Money |
|  |  | Economic impact |
|  |  | Lack of money, lack of preventive support |
|  | Motivation and attitiudes of staff | Motivation of staff |
|  |  | Attitude of workers |
|  |  | People’s carelessness and negligence |
|  |  | Employees' awareness, laziness, and beliefs |
|  |  | People’s careless attitude, limited awareness |
|  | Weather conditions | Climate (freezing or evaporation of disinfectant, etc.) |
|  |  | Weather |
|  | Unexpected events (e.g. power cuts, etc.) | Unexpected events (e.g. power cuts, etc.) |
|  |  | Force majeure (e.g. power cut, etc.) |
| 6. Stakeholders involved in African swine fever control | Laboratories | Laboratories |
|  |  | Veterinary laboratories |
|  | Pigkeepers | Pigkeepers |
|  |  | Pigkeepers (owners) |
|  |  | Farm owners |
|  |  | Farmers |
|  |  | Farms |
|  | Breeding association | Breeding associations incl. other pig farmers |
|  |  | Pigs Breeding Cooperative |
|  | Farm workers | Pig workers |
|  |  | Farm workers |
|  | Animal-waste processing plant | Animal waste disposal |
|  |  | Animal disposal |
|  | Farm veterinarians | Farm veterinarians |
|  |  | Veterinarians |
